# Edaphobacter paludis sp. nov., a new acidophilic representative of the Acidobacteriota isolated from fen soils

**DOI:** 10.1099/ijsem.0.006500

**Published:** 2024-08-28

**Authors:** Katharina J. Huber, János Papendorf, Carolin Pilke, Boyke Bunk, Cathrin Spröer, Sarah Kirstein, Jacqueline Wolf, Meina Neumann-Schaal, Manfred Rohde, Michael Pester

**Affiliations:** 1 Department of Microorganisms, Leibniz Institute DSMZ – German Collection of Microorganisms and Cell Cultures, Braunschweig, Germany; 2 Bioinformatic Services, Leibniz Institute DSMZ – German Collection of Microorganisms and Cell Cultures, Braunschweig, Germany; 3 Department of Metabolomics & Services, Leibniz Institute DSMZ – German Collection of Microorganisms and Cell Cultures, Braunschweig, Germany; 4 Braunschweig Integrated Centre of Systems Biology (BRICS), Braunschweig, Germany; 5 Department of Medical Microbiology, Central Facility for Microscopy, Helmholtz Centre for Infection Research, Braunschweig, Germany; 6 Institute of Microbiology, Technische Universität Braunschweig, Braunschweig, Germany

**Keywords:** acidobacteria, fen soils, peat, soil bacteria

## Abstract

Two new strains JP48^T^ and JP55 affiliated with the acidobacterial class *Terriglobia* have been isolated from fen soil sampled in the Fichtelgebirge Mountains near Bayreuth, Germany. Both strains were Gram-stain-negative, non-motile, non-spore-forming rods that divide by binary fission, segregate exopolysaccharide-like material and form capsules. Strains JP48^T^ and JP55 grew at 4–36 °C (optimum at 27 °C), pH 3.6–7.3 (optimum at pH 4.6–5.5) and with NaCl concentrations of 0–3% (optimum at 1.0%; w/v). Strains JP48^T^ and JP55 grew aerobically on a wide range of organic substrates including mono- and oligosaccharides, amino acids and short-chained fatty acids. MK-8 was identified as the major respiratory quinone. The major fatty acids for strains JP48^T^ and JP55 were *iso*-C_15 : 0_, C_16 : 1_ ω7c, C_16 : 0_ and *iso*-diabolic acid. Phosphatidylglycerol, phosphatidylethanolamine, diphosphatidylglycerol, lysophophatidylethanolamine, phosphatidylcholine, unidentified glyco- and glycophospholipids, and unidentified high mass lipid species were the major polar membrane lipids. The G+C content of strains JP48^T^ and JP55 was 57.4 and 57.2 mol%, respectively. The genomes of strains JP48^T^ and JP55 contained nine potential secondary metabolite regions encoding for the compound classes NRPS(-like), T3PKS, terpene, or lanthipeptide class IV. Phylogenetic reconstruction and 16S rRNA gene sequence similarities of 98.3 and 96.9% identified *Edaphobacter dinghuensis* DHF9^T^ and *Edaphobacter lichenicola* DSM 104462^T^ as the most closely related type strains to strains JP48^T^ and JP55. Based on their phenotype, phylogeny and chemotaxonomy, we propose the novel species *Edaphobacter paludis* sp. nov. (type strain JP48^T^=DSM 109919^T^=CECT 30269^T^; additional strain JP55=DSM 109920=CECT 30268) within the class *Terriglobia* of the phylum *Acidobacteriota*.

## Data Summary

All supporting data have been provided within the article or through supplementary data files.

## Introduction

Members of the phylum *Acidobacteriota* typically represent 5–60% of the bacterial community in different soil environments worldwide [1,5] including peat bogs and fens [6,9]. Formerly classified into 26 subdivisions (SDs) [10], the *Acidobacteriota* are currently subdivided into 15 class-level units, five of which contain described members [11]. Of those 15 class-level units, 13 are currently also mirrored by the Genome Taxonomy Database [12,13]. Acidic *Sphagnum*-dominated peatlands are typically dominated by members of the class *Terriglobia* ([14]; formerly class ‘Acidobacteria’ Trash and Coates 2010) with members of the *Terriglobales* (formerly SD1), *Bryobacterales* (formerly SD3), and uncultured SD2 constituting the major order-level taxa (relative abundance in decreasing order) [6,7, 15, 16]. Up to date, a comparably low number of 79 species with validly published names spanning 30 genera within the *Acidobacteriota* have been successfully isolated in pure culture (www.lpsn.dsmz.dehttps://lpsn.dsmz.de/phylum/acidobacteriota). Most of the currently known isolates belong to the 15 genera within the class *Terriglobia* [14] and originate from different peatland and upland soil environments, including the genera *Granulicella* [17], *Telmatobacter* [18], *Edaphobacter* [19] and *Occallatibacter* [20]. In the present study, we describe two novel isolates that represent a novel species of the genus *Edaphobacter* within the class *Terriglobia*. These two strains are the first isolates within the genus *Edaphobacter* that were isolated from fen soil.

## Origin and isolation

Two new acidobacterial strains (JP48^T^ and JP55) were isolated from fen soil sampled at 10–20 cm depth. The soil sample was collected from the Schlöppnerbrunnen II fen (pH 4–5; 50° 8′ 8.27″ N 11° 52′ 48.3″ E) close to Bayreuth, Germany on 28 November 2018, transported directly to the laboratory, and stored at 4 °C until further usage. To set up an enrichment, a soil suspension containing 5 g fen soil and 49.5 ml MES buffer (10 mM, pH 5.0) was serially diluted in 1 : 10 steps and subsequently inoculated in soil solution equivalent (SSE)/HD 1 : 10 medium (DSMZ medium 1426 [21]). Cultivation was performed in liquid medium or in medium solidified with purified agar under three different conditions [oxic, micro-oxic (8–10% O_2_; candle jar technique), and alternating oxygen conditions: 1 week under oxic and 1 week under micro-oxic conditions]. The SSE/HD 1 : 10 medium was based on SSE [22], supplemented with 10-vitamin solution [23], a trace element solution SL-10 [24] and, if indicated, solidified with purified agar (Oxoid). In the liquid approach, 96-well plates containing 180 µl SSE/HD 1 : 10 medium (pH 5.0) were mixed with 20 µl of the respective dilutions (10^−2^-10^−9^) of the soil suspension and incubated at 20 °C in the dark under oxic, micro-oxic, or alternating oxygen conditions. Additionally, 100 µl of the serial dilution step 10^−3^ were equally spread on SSE/HD 1 : 10 medium plates (pH 5.0), incubated at 20 °C in the dark under oxic, micro-oxic, or alternating oxygen conditions as well. After 4 weeks of incubation, turbid wells of the 96-well plates and grown colonies on the agar plates were screened for the presence of *Acidobacteriota* by an *Acidobacteriota*-specific PCR employing the primer pair Acido31f [25] and 1492r [26] following the PCR conditions laid out in the Supplementary Material. *Acidobacteriota* positive cultures were purified by subsequent subculturing on SSE/HD 1 : 10 medium plates under oxic conditions until two new strains JP48^T^ and JP55 were isolated from single colonies in pure culture. JP48^T^ was retrieved from a solid medium plate incubated under changing oxygen conditions while JP55 was isolated from a solid medium plate incubated under oxic conditions.

## Morphological and physiological characterization

On SSE/HD 1 : 10 agar plates, strains JP48^T^ and JP55 formed pale pink, transparent, shiny and smooth colonies with clear margins that were 0.5–1.0 mm in diameter. In liquid medium, strains JP48^T^ and JP55 formed pale pink biomass.

**Fig. 1.**
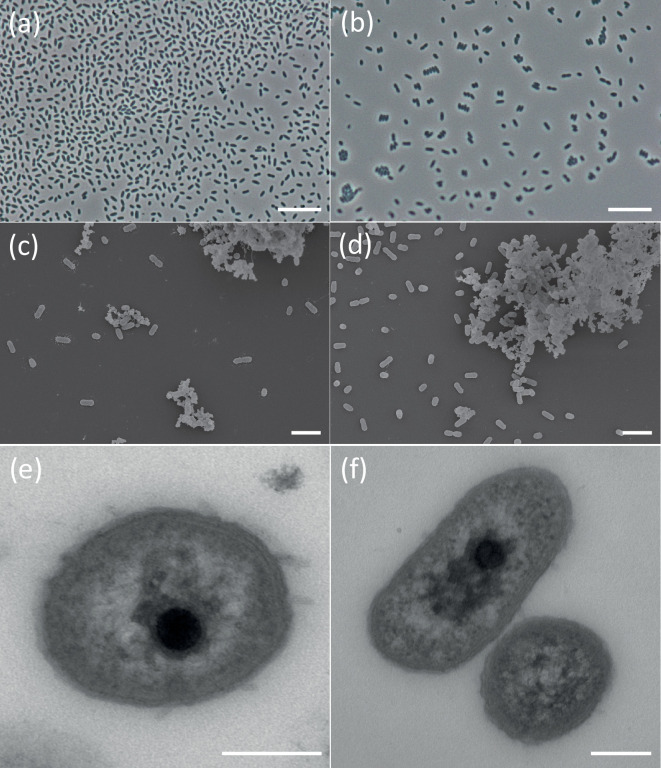
Phase-contrast microscopy images of strains JP48^T^ (**a**) and JP55 (**b**). Scale bar, 10 µm; scanning electron microscope pictures of strains JP48^T^ (**c**) and JP55 (**d**). Scale bar, 2 µm; transmission electron microscopy images of strains JP48^T^ (**e**) and JP55 (**f**). Scale bar, 200 nm.

For the evaluation of cell-wall structures, capsules, and endospores, the strains were stained according to the Gram, India ink and malachite green method, respectively, as described before [27], and subsequently examined by light microscopy (Zeiss Axio Scope A.1 equipped with an AxioCam MRc camera, Carl Zeiss). Strains JP48^T^ and JP55 were non-motile rods (0.30×1.10 µm and 0.40×0.75 µm wide and long, respectively) that divided by binary fission (Fig. 1a, b). The Gram-negative cell-wall structure of both strains was confirmed by transmission electron microscopy. Similar to other members of the *Acidobacteriota*, no endospores were detected. Formation of aggregates observed in liquid cultures was likely supported by extracellular polymeric substance-like material, as visible in the scanning (Fig. 1c, d) and transmission electron microscope images (Fig. 1e, f). Details of the transmission and scanning electron microscopy are given in the Supplementary Material. In addition, the presence of capsules was proven by India ink staining and light microscopy, thereby differentiating strains JP48^T^ and JP55 from all other known *Edaphobacter* strains.

Catalase and cytochrome c-oxidase activities were tested by standard protocols [28,29]. While JP48^T^ and JP55 showed positive catalase activity, no reaction of cytochrome c-oxidase was determined.

Anaerobic growth of strains JP48^T^ and JP55 was tested in duplicates on SSE/HD 1 : 10 medium pH 5.0 under anoxic conditions by the application of the Oxoid AnaeroGen Compact System. Despite an extended incubation time neither strain JP48^T^ nor strain JP55 formed biomass under anoxic conditions. The growth ranges and optima of strains JP48^T^ and JP55 in terms of temperature (4, 10, 15, 20, 22, 24, 27, 30, 36, 40 and 45 °C) and pH (pH 2.5–10; 0.5 pH unit steps) were tested in liquid SSE/HD 1 : 10 medium. While the pH of the medium for the temperature test was buffered at pH 5.0, the pH of the medium for the pH tolerance test was prepared using buffers MES (pK_S_ 6.15), HEPES (pK_S_ 7.48), HEPPS (pK_S_ 8.00) and CHES (pK_S_ 9.30) according to their optimal buffer range (10 mM final concentration). For the salinity tolerance test, NaCl concentrations of 0.00, 0.25, 0.50, 1.00, 3.00, 5.00, 7.50 and 10.00% (w/v) were adjusted in liquid HD 1 : 10 medium (DSMZ medium 1124). Strains JP48^T^ and JP55 grew between 4 and 36 °C and showed best growth at 15–36 °C (optimum 27 °C). JP48^T^ and JP55 tolerated pH values between pH 3.6 and 7.3 and grew optimally at pH 4.6–5.5. Both strains showed a broad NaCl tolerance and grew at 0–3% NaCl (optimum at 1.0%; w/v).

JP48^T^ and JP55 were incubated in triplicates in liquid 1×SSE solution to test their potential to use substrates such as sugars, keto acids, organic acids, amino acids, alcohols, casamino acids, laminarin, peptone, yeast extract, casein hydrolysate and Tween 80. The test medium was adjusted to pH 5.0, supplemented with 1 ml l^−1^ vitamin solution, 1 ml l^−1^ trace element SL-10 solution and 0.025% yeast extract (final concentration) and the respective test substrate (see Table S1, available in the online Supplementary Material, for applied substrate concentrations). After 8 weeks of incubation in the dark at 20 °C, growth as based on the tested substrates was determined by optical density measurements at 660 nm wavelength. Weak growth was defined as an increase in OD_660nm_ by a factor of 1.2–1.5 as compared to controls without substrates; for standard growth the threshold was set to a 1.5-fold increase in OD_660nm_. Strains JP48^T^ and JP55 grew on a large variety of different sugars, organic acids, and amino acids (Table S2; further details are given in the species description).

The degradation potential for different complex substrates was tested on solidified SSE medium supplemented with 1 ml l^−1^ vitamin solution, 1 ml l^−1^ trace element SL-10 solution, 0.005% yeast extract (w/v) and complex substrates such as starch, cellulose, pectin, chitin, polygalacturonic acid and xylan (0.5 g l^−1^ final concentration). After 4 weeks of incubations the degradation potential was detected by staining the agar with the respective staining solutions and the appearance of clearing zones as reported earlier [30]. In addition, APIZYM and API20NE galleries (bioMérieux) were employed to determine further exoenzyme activity potential, the formation of indole, aesculin degradation, urease activity, reduction of nitrogen and the assimilation capacity of different carbon compounds. Besides *Edaphobacter dinghuensis* DSM 29920^T^, as determined in the current study, JP48^T^ and JP55 were the only *Edaphobacter* strains to be able to reduce nitrate to nitrite under oxic conditions, which is the first step of aerobic nitrate reduction (Table S3).

## Phylogenetic placement

Colony PCR employing the primer pair 8f [31] and 1492r [26] enabled the amplification of the 16S rRNA genes of strains JP48^T^ and JP55, as described previously [30], with subsequent Sanger sequencing. Direct 16S rRNA gene sequence comparison by blast [32] identified *Edaphobacter dinghuensis* DHF9^T^ [33] and *E. lichenicola* DSM 104462^T^ [34] as the closest related type strains to strains JP48^T^ and JP55 (16S rRNA sequence identity of 98.3 and 96.9%, respectively). Strains JP48^T^ and JP55 displayed a 16S rRNA gene sequence similarity value of 99.9% to each other.

**Fig. 2.**
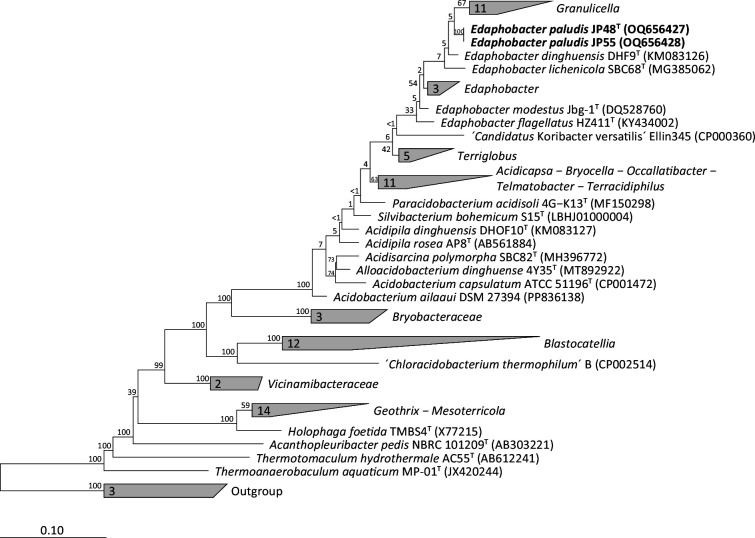
Maximum-likelihood phylogenetic tree based on almost full-length 16S rRNA gene sequences showing the relationships between strains JP48^T^, JP55 and related type strains. Bootstrap values are expressed as a percentages of 1000 replicates and are indicated at the respective branching points. The following sequences were used as outgroup: *Novipirellula rosea* LHWP3^T^ (JF748734), *Blastopirellula marina* DSM 3645^T^ (X62912) and *Pirellula staleyi* DSM 6068^T^ (CP001848). Bar indicates 10% nucleotide divergence.

For phylogenetic tree calculations, the 16S rRNA gene sequences of strains JP48^T^ and JP55 were added to the small subunit rRNA non-redundant reference database silva version 138.1 (www.arb-silva.de) via the program arb [35]. After the implemented automated alignment, the alignment was corrected manually based on the secondary structure information. Three different algorithms, namely neighbour-joining, maximum-parsimony and maximum-likelihood (termini filter; 1376 valid columns between position 63 and 1439 of the *Escherichia coli* 16S rRNA reference gene; 1000 bootstrap re-samplings), were employed for phylogenetic reconstruction. All three methods identified JP48^T^ and JP55 as members of the genus *Terriglobia* and confirmed the phylogenetic affiliation of strains JP48^T^ and JP55 with *E. dinghuensis* DHF9^T^ and *E. lichenicola* DSM 104462^T^ (Figs 2, S1 and S2).

## Phylogenomic placement

The genomes of strains JP48^T^ and JP55 were sequenced on a PacBio Sequel IIe instrument (Pacific Biosciences) as detailed in the Supplementary Material. The genomes of JP48^T^ and JP55 were 4 038 441 and 4 316 569 bp and their G+C contents were 57.4 and 57.2 mol%, respectively. A high sequencing coverage value was obtained for both strains (1764 and 1966-fold coverage, respectively) by PacBio sequencing, which was further refined by Illumina short-read sequencing (Supplementary Material). After the assembly, the genomes were automatically oriented after *oriC* and subsequently analysed with the prokka pipeline resulting in 3412 and 3696 CDS regions, three and three 16S rRNA genes, and 48 and 48 tRNAs for the strains JP48^T^ and JP55, respectively (Table S4).

**Fig. 3.**
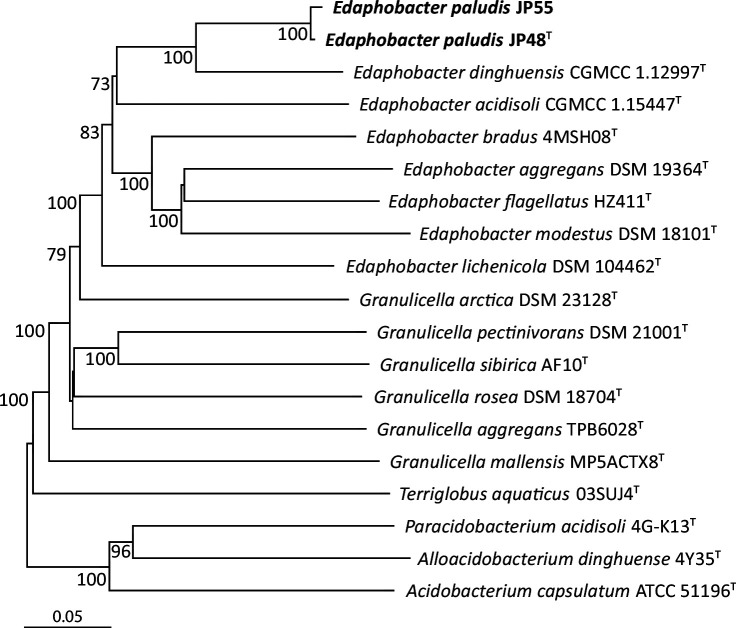
Whole-proteome-based GBDP distances tree for strains JP48^T^ and JP55. The tree was inferred with FastME 2.1.6.1 [36] and branch lengths are scaled via GBDP distance formula d5. Branch values are GBDP pseudo-bootstrap support values >60% from 100 replications, with an average branch support of 85.6%. The tree was midpoint-rooted [37].

To confirm the phylogenetically closest relatives to strains JP48^T^ and JP55 as identified by 16S rRNA gene sequence analysis, a proteome-based phylogenomic tree was calculated for the genomes on the TYGS webpage (Type Strain Genome server; https://tygs.dsmz.de). The Genome blast Distance Phylogeny (GBDP) was employed to calculate the phylogenetic distances with the algorithm 'coverage' and distance formula d5. Subsequently, the phylogenomic trees were calculated from whole-proteome and whole-genome sequence-based GBDP distances with FastME 2.1.6.1 [36], replicated with 100 pseudo-bootstraps, and finally rooted at the midpoint, respectively [37]. The phylogenomic trees showed *E. dinghuensis* CGMCC 1.12997^T^ as the phylogenetically closest relative to strains JP48^T^ and JP55 (Figs 3 and S3).

## Whole-genome similarity indices

Genome similarity indices of strains JP48^T^ and JP55 and their phylogenetically closest relatives were used for species and genus demarcation. Genome-wide average nucleotide identity (ANI), average amino acid identity (AAI) and digital DNA–DNA hybridization (dDDH) were calculated employing the OrthoANIu algorithm on the EzBioCloud webpage (www.ezbiocloud.net/tools/ani [38]), the online AAI calculator (http://enve-omics.ce.gatech.edu/ [39]) and the Genome-to-Genome Distance calculator version 3.0 (https://ggdc.dsmz.de/ggdc.php; [40]), respectively. ANI and dDDH values of 99.6 and 97.7% confirmed that strains JP48^T^ and JP55 belong to the same species as the values are above the recommended thresholds of 95 and 70% for species delineation, respectively [41,42]. However, the ANI and dDDH values of 80.7–80.8 and 24.2% for JP48^T^ and JP55 in comparison to *E. dinghuensis* CGMCC 1.12997^T^ and of 74.3 and 20.3% in comparison to *E. lichenicola* DSM 104462^T^ support that strains JP48^T^ and JP55 represent a novel species within the genus *Edaphobacter*. The AAI values of strains JP48^T^ and JP55 in comparison with their phylogenetically closest relatives *E. dinghuensis* CGMCC 1.12997^T^ and *E. lichenicola* DSM 104462^T^ were above the genus demarcation threshold of 65% (83.9–84.2% and 71.1–71.4%; Table S5 [43]), supporting the inclusion of both strains into the genus *Edaphobacter*.

## Genomic features

The annotation and analysis platform implemented on the Bacterial and Viral Bioinformatics Resource Centre (BV-BRC) webpage assigned 29 and 27% of the genome sequences of strains JP48^T^ and JP55 to known subsystem categories, respectively. The groups encompassing most of the assigned genes were ‘protein synthesis’ (131 and 128 genes for strains JP48^T^ and JP55, respectively), ‘cofactors, vitamins, prosthetic groups’ (86 and 87), ‘amino acids and derivates’ (76 and 76), and ‘stress response, defence and virulence’ (64 and 65). While most of the known and strongly represented subsystem categories contained genes for the primary dissimilatory and assimilatory metabolism, elevated gene numbers in the category ‘stress response, defence and virulence’ would explain the broad temperature and pH tolerance ranges detected for strains JP48^T^ and JP55. This likely reflects the varying temperature [44] and pH [45,46] conditions in the fen soil across the seasons from which strains JP48^T^ and JP55 were isolated.

The potential for antibiotics resistance was encoded by 31 genes in the case of JP48^T^ and JP55. Additionally, the genomes of strains JP48^T^, JP55, *E. dinghuensis* CGMCC 1.12997^T^ (NCBI RefSeq assembly GCA_014640335.1) and *E. lichenicola* DSM 104462^T^ (NCBI RefSeq assembly GCA_025264645.1) were analysed via the antiSMASH pipeline (https://antismash.secondarymetabolites.org [47]). The options ‘relaxed detection strictness’ and ‘all possible extra features’ detected nine secondary metabolite regions for strains JP48^T^ and JP55 and eight secondary metabolite regions for *E. dinghuensis* CGMCC 1.12997^T^ and *E. lichenicola* DSM 104462^T^, respectively, encoding for either T3PKS, NRPS(-like), T1PKS, terpene, lanthipeptide class IV, RRE-containing, lassopeptide, RiPP-like or redox-cofactor (Table S6). Three clusters identified in the genomes of the strains JP48^T^ and JP55 showed similarity values of 5–11% to the next most similar known cluster and two clusters identified in the genome of *E. dinghuensis* CGMCC 1.12997^T^ displayed similarity values of 20–50% to the next most similar known cluster. However, no cluster identified in the genome of *E. lichenicola* DSM 104462^T^ could be addressed to any known secondary metabolite cluster.

Genes for the degradation of complex carbon compounds such as xylan (endo-1,4-beta xylanase EC 3.2.1.8) and laminarin (laminarinase EC 3.2.1.39), as detected in the genomes of the two strains, indicate that strains JP48^T^ and JP55 are able to use complex hemicellulose as a growth substrate. However, this ability could not be proven under laboratory conditions.

## Chemotaxonomic characterization

To determine fatty acids, including membrane-spanning lipids, of the all studied strains, cells of strains JP48^T^, JP55, *Edaphobacter dinghuensis* DSM 29920^T^, *E. lichenicola* DSM 104462^T^, *E. modestus* DSM 18101^T^, and *E. aggregans* DSM 19364^T^ were grown on SSE/HD 1 : 10 medium (pH 5.5) at 25 °C for 7 days (Table 1). About 100 mg wet biomass was extracted based on the protocol described by Damsté *et al*. [48] using chloroform instead of dichloromethane. The extracted fatty acids were converted to fatty acid methyl esters (FAMEs) and analysed via a GC coupled to a flame ionization detector (on an Agilent Technologies 6890B) according to the protocol described by Sasser [49]. Equivalent chain length values were calculated analogously to the Sherlock Microbial Identification System to provide the peak naming according to the TSBA6 database. Identification of fatty acids was performed via MS [50]. The position of double bonds of monosaturated fatty acids was determined by a derivatization with dimethyl sulfide [51]. Additionally, the FAME mixtures were silylated with *N*-methyl-*N*-(trimethylsilyl)trifluoroacetamide and measured via GC-MS to investigate glycerol bond fatty acids.

**Table 1.** Cellular fatty acid composition of strains JP48^T^, JP55 and described *Edaphobacter* species type strains Strains: 1, JP48^T^; 2, JP55; 3, *Edaphobacter dinghuensis* DSM 29920^T^; 4, *E. lichenicola* DSM 104462^T^; 5, *E. modestus* DSM 18101^T^; 6, *E. aggregans* DSM 19364^T^. All data were obtained during this study. All strains were grown on SSE/HD 1 : 10 medium at 25 °C for 7 days. The values are given as percentages. –, Not detected. As discussed by Belova *et al*. [34], *E. acidisoli* 4 G-K17^T^ [52], *E. flagellatus* HZ411^T^ [55] and *E. bradus* 4MSH08^T^ [55] are not included in the comparison as the procedure used for the analysis of lipids does not allow detection of *iso*-diabolic acid and the strains were not available for analysis in this study. However, their other common fatty acids are also *iso*-C_15 : 0_, C_16 : 1_ ω7c and C_16 : 0_.

Fatty acid	1	2	3	4	5	6
C_12 : 0_	0.4	–	–	0.3	–	–
*iso-*C_13 : 0_	–	–	–	1.6	–	–
C_14 : 0_	1.3	0.9	0.9	1.8	1.6	1.6
C_14 : 1_ ω5c	0.2	0.3	0.3	0.3	0.7	0.6
C_15 : 0_	0.3	0.2	0.3	1.2	0.2	0.4
*iso-*C_15 : 0_	**33.2**	**32.8**	**36.1**	**25.5**	**20.5**	**28.0**
*anteiso-*C_15 : 0_	0.4	0.2	–	–	–	–
dimetyhl-C_15 : 0_ A*	0.2	0.2	0.4	–	–	–
dimethyl-C_15 : 0_ B*	0.2	0.2	0.4	–	–	–
C_15 : 1_ ω6c	0.1	0.2	0.4	–	0.2	0.3
C_16 : 1_ ω7c	**15.1**	**17.8**	**11.6**	**20.2**	**31.4**	**22.5**
C_16 : 1_ ω7t	0.8	0.6	0.8	–	0.3	0.2
C_16 : 0_	**10.2**	9.7	7.4	**21.3**	9.0	**11.2**
*iso-*C_16 : 0_	0.3	0.2	0.1	0.2	0.2	0.5
C_17 : 0_	0.7	0.7	0.7	2.2	0.5	0.8
*iso-*C_17 : 0_	1.5	1.1	1.1	1.0	1.1	2.2
*anteiso-*C_17 : 0_	0.6	0.6	0.1	0.4	0.2	0.3
*iso-*C_17 : 1_ ω7c	0.6	0.4	0.4	0.7	0.6	1.3
C_17 : 0_ cyclo ω7c	3.3	3.0	5.8	–	–	–
C_18 : 0_	4.2	2.1	1.6	6.0	2.0	2.6
C_18 : 1_ ω7c	–	0.2	0.1	0.3	0.1	–
C_18 : 1_ ω9c	–	1.4	2.2	3.5	0.4	0.9
C_20 : 0_	1.3	1.2	1.0	1.8	0.6	0.7
*iso*-diabolic acid	**25.0**	**26.2**	**28.3**	**11.8**	**30.4**	**26.4**

a *Too small for unambiguous identification.

The fatty acid compositions of strains JP48^T^ and JP55 resembled those of the known *Edaphobacter* strains (Table 1). *Iso*-C_15 : 0_ (32.8–33.2%), C_16 : 1_ ω7c (15.1–17.8%), C_16 : 0_ (9.7–10.2%) and *iso*-diabolic acid (25.0–26.2%) were identified as the major fatty acids. Interestingly, the presence of a cyclic C_17 : 0_ fatty acid was also determined for strains JP48^T^ (3.3%) and JP55 (3.0%); this fatty acid was determined in just two other type strains within the genus *Edaphobacter*, namely *Edaphobacter dinghuensis* DSM 29920^T^ (5.8%) and *E. acidisoli* 4 G-K17^T^ [52]. Other than described by Damsté *et al*. [48], no glycerol ethers of *iso*-diabolic acid or similar ethers could be detected, which could be attributed to the different growth conditions in the two studies. It is known that different media, growth conditions and the age of cultures influence the fatty acid composition [51]. Accordingly, different conditions can lead to compounds falling below the detection limit.

For respiratory quinones analyses, cells of strains JP48^T^ and JP55 were grown in SSE/HD 1 : 10 medium (pH 5.5) at 20 °C for 10 days. Respiratory quinones were extracted from 5 mg wet biomass via solid-phase extraction and analysed via reversed phase HPLC coupled to a diode array detector [50]. MK-8 was detected as the sole respiratory quinone for both strains.

For the detection of membrane polar lipids, the strains were grown according their optimum growth conditions. JP48^T^, JP55 and *E. dinghuensis* DSM 29920^T^ on DSMZ medium 1426 at 28 °C, *E. lichenicola* DSM 104462^T^ on DSMZ medium 1284 at 25 °C, *E. modestus* DSM 18101^T^ on DSMZ medium 1124 at 25 °C, and *E. aggregans* DSM 19364^T^ on DSMZ medium 1135 at 20 °C. Membrane polar lipids of all the strains were extracted from lyophilized biomass using a modified Bligh and Dyer extraction [53]. Samples were extracted twice with methanol:dichlormethane (DCM):0.3% NaCl (2 : 1 : 0.8, v/v/v) by ultrasonication for 10 min. Combined DCM phases were additionally extracted twice with 0.3% NaCl and evaporated under a stream of nitrogen. Dried extracts were reconstituted in hexane:isopropanol:water (718 : 271 : 10, v/v/v) and filtered through a regenerated cellulose syringe filter (Minisart RC4, Sartorius). Membrane polar lipids were analysed by HPLC-MS as described previously [54]. The polar lipid profile of strains JP48^T^ and JP55 consisted of phosphatidylethanolamine, phosphatidylglycerol, diphosphatidylglycerol, phosphatidylcholine and lysophosphatidyl-ethanolamine. In addition, unidentified glyco- and glycophospholipids could be detected as well as several unidentified high mass lipid species (Table S7). High mass intact polar lipids were already described to be present in large amounts in *E. lichenicola* SBC^T^ [34] and were detected in all *Edaphobacter* strains analysed in this study.

Based on the presented morphological, physiological and phylogenetic characteristics, strains JP48^T^ and JP55 represent a new species within the acidobacterial genus *Edaphobacter* named *Edaphobacter paludis* sp. nov. (Table 2).

**Table 2.** Characteristics of JP48^T^ and JP55 as compared with their phylogenetically most closely related species type strains Strains: 1, JP48^T^; 2, JP55; 3, *Edaphobacter dinghuensis* DHF9^T^ [33]; 4, *E. lichenicola* SBC68^T^ [34]; 5, *E. modestus* Jbg-1^T^ [19]; 6, *E. aggregans* Wbg-1^T^ [19]; 7, *E. acidisoli* 4 G-K17^T^ [54]; 8, *E. bradus* 4MSH08^T^ [55]; 9, *E. flagellatus* HZ411^T^ [55]. Polar lipids of strains 1–6 were analysed in this study.

Characteristics	1	2	3	4	5	6	7	8	9
Source	Fen soil	Fen soil	Subtropical forest soil	Thalli of *Cladonia* sp.	Alpine soil	Deciduous forest soil	Forest soil	Forest soil	Forest soil
Cell shape	Rod	Rod	Rod	Rod/cell rosettes	Rod	Rod	Rod	Rod	Rod
Cell length×width (µm)	0.75.–1.1×0.3–0.4	0.75.–1.1×0.3–0.4	0.8–1.4×0.5–0.7	1.3–2.7×0.4–0.7	1.0–1.8×0.5–0.7	1.5–2.1×0.7–0.9	1.1–1.5×0.3–0.5	1.3–1.8×0.6–0.9	1.0–1.6×0.4–0.8
Cell division	BF	BF	BF	BF	BF	BF	BF	BF	BF
Motility	−	−	−	−	+	−	−	−	+
Pigmentation	Pale pink	Pale pink	Light beige	Pinkish	Light beige	Light beige	Beige	Canary yellow	Pale yellow
Capsule/EPS formation	+	+	−	−	−	−	−	−	−
Oxidase	−	−	−	+	+	−	+	−	−
Catalase	+	+	−	−	+	+	+	+	−
Quinone	MK-8	MK-8	nd	MK-8	nd	nd	nd	nd	nd
NaCl tolerance for growth (%, w/v)	0–3	0–3	0–2	0–1	nd	nd	0–2.5	0–3	0–2.5
Temperature range (optimum) for growth (°C)	4–36 (15–36)	4–36 (15–36)	10–33 (28–33)	7–37 (20–30)	15–30 (30)	15–37 (30)	10–42 (28)	10–37 (28)	10–37 (28)
pH range (optimum) for growth	3.6–7.3 (4.6–5.5)	3.6–7.3 (4.6–5.5)	3.5–5.5 (4.0)	3.4–7.0 (4.3–5.6)	4.5–7.0 (5.5)	4.0–7.0 (5.5)	3.0–7.0 (4.0–5.5)	3.0–6.5 (4.0–5.5)	3.5–6.5 (4.5–5.5)
DNA G+C-content (mol %)	57.4	57.2	57.7	54.7	55.8	56.9	57.6	59.6	57.7
Lipids	PE, PG, LPE, PC, DPG, GPL, GL, high mass IPLs	PE, PG, LPE, PC, DPG, GPL, GL, high mass IPLs	PE, PG, PC, DPG, GPL, high mass IPLs	PE, PG, LPE, PC, DPG, GPL, GL, high mass IPLs	PE, PC, DPG, GPL, GL, high mass IPLs	PE, LPE, PC, OL, DPG, GPL, GL, high mass IPLs	PE, PG, APL, PL	PE, PDE, GL	PE, PDE, GL

+, positive; –, negative; nd, no data available. BF, binary fission; EPS, exopolysaccharide-like material; DPG, diphosphatidylglycerol; PE, phosphatidylethanolamine; PC, phosphatidylcholine; PG, phosphatidylglycerol; LPE, lysophosphatidylethanolamine; PDE, phosphatidyldimethylethanolamine; IPL, intact polar lipid; PL, unidentified phospholipid; APL, unidentified aminophospholipid; GL, unidentified glycolipid; GPL, unidentified glycophospholipid; OL, ornithine lipid.

## Description of *Edaphobacter paludis* sp. nov.

*Edaphobacter paludis* (pa.lu’dis. L. gen. n. *paludis*, of a marsh).

Cells are aerobic rods, 0.3–0.4 µm wide, 0.75–1.10 µm long, and divide by binary fission. No spores but capsules are formed. Exopolysaccharide-like material is present. No motility has been observed. Catalase-positive but cytochrome-c- and oxidase-negative. Colonies on plates are 0.5–1.0 mm in diameter, pale pink pigmented, transparent, shiny and smooth with clear margins. In liquid cultures, pale pink cell aggregates are formed that can be dissolved by shaking.

Grows at 4–36 °C (best at 15–36 °C, optimum at 27 °C), pH 3.6–7.3 (best at pH 4.6–5.5) and NaCl concentrations of 0–3% (best at 1.0%; w/v).

The identified major fatty acids are *iso-*C_15 : 0_, C_16 : 1_ ω7*c*, C_16 : 0_ and *iso*-diabolic acid. In addition, C_17 : 0_ cyclo ω7c also occurs. MK-8 is the sole respiratory quinone. The major polar lipids are phosphatidylglycerol, phosphatidylethanolamine, diphosphatidylglycerol, lysophophatidylethanolamine and phosphatidylcholine, next to unidentified glyco- and glycophospholipids as well as unidentified high mass lipid species.

Grows aerobically on glucose, lactose, fructose, cellobiose, galactose, mannose, melezitose, raffinose, maltose, rhamnose, sucrose, trehalose, xylose, *myo*-inositol, casamino acids, casein-hydrolysate, yeast extract, glycerin, peptone, *N*-acetylglucosamine, *N*-acetylgalactosamine, arabinose, 2-oxogluconate, 1,2-butanediol, 2,3-butanediol, 1,2-propanediol, alanine, arginine, asparagine, glutamine, l-isoleucine, ornithine, proline, tryptophan, acetate, butyrate, formate, β-hydroxybutyrate, γ-hydroxybutyrate, isobutyrate, tyrosine, serine, phenylalanine, glycine, leucine, histidine, valine, methionine, threonine, succinate, nicotinic acid, Tween 80, adipate, protocatechuate, shikimate, aspartate, glutamate, laminarin, malate, citrate, tartrate, fermented rumen extract, isovaleric acid, heptanoic acid, fumarate, trimethoxybenzoate, crotonate, 2-oxoglutarate, Na–pyruvate, acetoin, ascorbate and glyoxylate. Weak growth occurs on 2-oxovalerate, benzoate, cysteine, maleic acid, lyxitol, glucosamine, gluconate, caproate, caprylate, dulcitol, ethylene glycol, erythrose, erythrulose, α-hydroxybutyrate, isocitrate and levulinate. No growth could be determined on fucose, sorbose, lyxose, adenitol, arabitol, mannitol, sorbitol, xylitol, l-lysine–HCl, hydroxy-l-proline, glycolate, malonate, propionate, oxaloacetate, lactate, butanol, ethanol, methanol, propanol and glucuronate. Growth under anoxic conditions was not observed.

Positive for the following enzymes: acid phosphatase, α-galactosidase, β-galactosidase (APIZYM and API20NE), α-glucosidase, β-glucosidase (APIZYM and API20NE), *N*-acetyl-β-glucoseamidase and α-fucosidase. Activity of naphthol-AS-BI-phosphohydrolase and β-glucuronidase varies from weak positive (strain JP55) to positive (strain JP48^T^). Nitrate is (weakly) reduced to nitrite. A varying efficiency of starch hydrolysis could be detected in strain JP55. Degradation of every other investigated polymeric substance was negative.

The type strain is JP48^T^ (=DSM 109919^T^=CECT 30269^T^) and a second strain of the species is JP55 (=DSM 109920=CECT 30268). Both were isolated from fen soil collected in Schlöppnerbrunnen II in Germany. The DNA G+C content of the type strain is 57.4 mol% and the GenBank/EMBL/DDBJ accession numbers for the 16S rRNA gene sequence and for the genome are OQ656427/OQ656428 and CP121194/CP121195, respectively.

## supplementary material

10.1099/ijsem.0.006500 Uncited Supplementary Material 1.

## Abbreviations

AAI: average amino acid identity
ANI: average nucleotide identity
APL: unidentified aminophospholipid
BF: binary fission
BV-BRC: Bacterial and Viral Bioinformatics Resource Centre
CHES: N-cyclohexyl-2-aminoethanesulfonic acid
dDDH: digital DNA-DNA hybridization
DPG: diphosphatidylglycerol
EPS: exopolysaccharide-like material
FAME: fatty acid methyl ester
(GC)-MS: (Gas chromatography–)mass spectrometry
GL: unidentified glycolipid
GPL: unidentified glycophospholipid
HD: yeast glucose
HEPES: 4-(2-hydroxyethyl)-1-piperazineethanesulfonic acid
HEPPS: 3-[4-(2-Hydroxyethyl)piperazin-1-yl]propane-1-sulfonic acid
HPLC: High-performance liquid chromatography
IPL: intact polar lipid
LPE: lysophosphatidylethanolamine
MES: 2-(N-morpholino)ethanesulfonic acid
OL: ornithine lipid
PC: phosphatidylcholine
PDE: phosphatidyldimethylethanolamine
PE: phosphatidylethanolamine
PG: phosphatidylglycerol
PL: unidentified phospholipid
SD: subdivision
SSE: Soil solution equivalent
