# Supplementary material for: Edaphobacter paludis sp. nov., a new acidophilic representative of the Acidobacteriota isolated from fen soils
Source: Int J Syst Evol Microbiol. 2024 Aug 28;74(8):006500. doi: 10.1099/ijsem.0.006500 (PMC11354219; doi:10.1099/ijsem.0.006500)
Supplement: Uncited Supplementary Material 1. [file ijsem-74-06500-s001.pdf]

Supplementary Material

***Edaphobacter paludis* sp. nov. – a new acidophilic representative of the *Acidobacteriota* isolated from fen soils**

Katharina J. Huber[1†]*, János Papendorf[1†], Carolin Pilke[1], Boyke Bunk[2], Cathrin Spröer[2], Sarah Kirstein[3], Jacqueline Wolf[3], Meina Neumann-Schaal[3,4], Manfred Rohde[5], Michael Pester[1,6]

[1] Department of Microorganisms, Leibniz Institute DSMZ – German Collection of Microorganisms and Cell Cultures, Braunschweig, Germany

[2] Bioinformatic Services, Leibniz Institute DSMZ – German Collection of Microorganisms and Cell Cultures, Braunschweig, Germany

[3] Department of Metabolomics & Services, Leibniz Institute DSMZ – German Collection of Microorganisms and Cell Cultures, Braunschweig, Germany

[4] Braunschweig Integrated Centre of Systems Biology (BRICS), Braunschweig, Germany

[5] Department of Medical Microbiology, Central Facility for Microscopy, Helmholtz Centre for Infection Research, Braunschweig, Germany

[6] Institute of Microbiology, Technische Universität Braunschweig, Braunschweig, Germany

Keywords: Acidobacteria, soil bacteria, fen soils, peat

Repositories: The GenBank/EMBL/DDBL accession numbers for the 16S rRNA and draft genome sequences of the strains JP48^{T} and JP55 are OQ656427, OQ656428 and CP121194 and CP121195, respectively.

Data summary:

All supporting data have been provided within the article of through supplementary data files.

[†] Shared first authorship.

* Correspondence: K. Huber, Leibniz-Institute DSMZ – German Collection of Microorganisms and Cell Cultures GmbH, Inhoffenstraße 7B, 38124 Braunschweig, Germany.

Tel.: +49-0531-2616-365; Fax: +49-531-2616-415; Email: Katharina.Huber@dsmz.de

Material and Methods – *Acidobacteriota* specific colony PCR

Grown colonies on the solid medium and turbid wells in liquid medium approach were screened for the presence of *Acidobacteriota* by *Acidobacteriota* specific colony PCR. The respective PCR mixture included 16,94 µl PCR-water, 2 µl PCR buffer (10x), 0.4 µl dNTPs (10 mM each), 0.4 µl BSA (20 mg/ml), 0.08 µl forward primer [Acido31f (50 pmol/µl)], 0.08 µl reverse primer [1492r (50 pmol/µl)], 0.1 µl Taq-Polymerase (5 U/µl) and 1.0 µl DNA.

The DNA was subsequently amplified by the *Acidobacteriota* specific colony PCR programme including an initial denaturation step at 94°C for 10 min and 8 cycles of a touchdown PCR step [94°C for 30s, 60-56°C (each cycle the annealing temperature was decreased by 0.5°C) for 30 s and 72°C for 45 s]. Then 32 cycles of 94°C for 30s, 56°C for 30 s and 72°C for 45 s followed. The PCR reaction was finalized by an elongation step at 72°C for 7 min and a cooldown phase at 7°C for eternity.

Material and Methods – Transmission electron microscopy

For transmission electron microscopy (TEM) samples were fixed with 2% glutaraldehyde in culture medium, left for 30 min on ice, then further fixed with 5% formaldehyde and left for 5 h at 7°C. Then the samples were washed twice with 0.1 M HEPES buffer and immobilized with 2% water agar. Dehydration was achieved with a graded series of ethanol (10%, 30%, 50%) for 30 min on ice. The 70% ethanol step containing 2% uranyl acetate was performed overnight at 7°C, followed by the 90% ethanol step for 30 min on ice. The 100% ethanol step was performed twice for 30 min at room temperature. For embedding LRWhite resin (hard formular) was used. Polymerization of the LRWhite was carried out at 50°C for 2 days. Ultrathin sections were cut with a diamond knife and collected with a butvar coated copper grid. Post-staining of sections was done with 2% aqueous uranyl acetate for 3 min. After washing in distilled water and air-drying samples were examined in an EM 910 transmission electron microscope (Zeiss, Oberkochen, Germany) at an acceleration voltage of 80 kV. Images were taken at calibrated magnifications using a line replica and recorded digitally with a Slow-Scan CCD-Camera (ProScan, 1024x1024, Scheuring, Germany) applying the ITEM-Software (Olympus Soft Imaging Solutions, Münster, Germany).

Material and Methods – Field emission scanning electron microscopy

For field emission scanning electron microscopy (FESEM) HEPES buffer washed samples as described above were adsorbed onto poly-L-lysine coated cover slips (12 mm in diameter) for 10 min, fixed with 1% glutaraldehyde in TE buffer and washed twice with HEPES buffer. Samples were then dehydrated with a graded series of acetone (10, 30, 50, 70,90, 100%) for 10 min each step on ice. The 100% acetone step was repeated at room temperature before samples were critical point dried with liquid CO_2 (Leica, CPD 300) and sputter coated with gold-palladium (Bal-Tec, SCD 500). Samples were examined in a Zeiss Merlin field emission scanning electron microscope at an acceleration voltage of 5 kV using the SE

(secondary electron)-lens and Everhart-Thornley SE-detector in a 75:25 ratio. Images were recorded applying the SmartSEM software version 6.06.

Material and Methods – Genome sequencing

For the genome sequencing of the strains JP48^{T} and JP55 genomic DNA extraction was carried out on MasterPure^{TM} Gram Positive DNA Purification Kits from Epicentre®Biotechnologies Germany according to the manufacturer´s instructions. SMRTbell™ template libraries were prepared according to the instructions from PacificBiosciences, Menlo Park, CA, USA, following the Procedure & Checklist – Preparing Multiplexed Microbial Libraries Using SMRTbell® Express Template Prep Kit 2.0. Briefly, for preparation of 10 kb libraries 1 µg genomic DNA was sheared using g-tubes™ from Covaris, Woburn, MA, USA according to the manufacturer´s instructions. DNA was end-repaired and ligated to barcoded adapters applying components from the SMRTbell Express Template Prep Kit 2.0 from Pacific Biosciences, Menlo Park, CA, USA. Reactions were carried out according to the manufacturer´s instructions. Samples were pooled according to the calculations provided by the Microbial Multiplexing Calculator. Conditions for annealing of sequencing primers and binding of polymerase to purified SMRTbell™ template were assessed with the Calculator in SMRT®link, PacificBiosciences, Menlo Park, CA, USA. Libraries were sequenced on the Sequel*II* (PacificBiosciences, Menlo Park, CA, USA) taking one 15 h movie per SMRT cell. In the case of JP48^{T} and JP55 libraries for sequencing on Illumina platform were prepared applying Nextera XT DNA Library Preparation Kit with modifications [1] and sequenced on the Illumina NextSeq™ 550 (Illumina, San Diego, USA). For both strains long read genome assembly was performed with the "Microbial Assembly" protocol included in SMRTlink version 8 using default parameters with exception of the target genome size, which was set to 4.1 Mbp. The chromosomal contig was circularized, particularly artificial redundancies at the ends of the contigs were removed and adjusted to *dnaA*. Identification of redundancies and the replication genes has been done based on BLAST, circularization and rotation to the replication genes has been performed by genomecirculator.jar tool (https://github.com/boykebunk/genomefinish). Additionally, in the case of the strains JP48^{T} and JP55 error-correction was performed by a mapping of Illumina short reads onto finished genome using Burrows-Wheeler Alignment bwa 0.6.2 in paired-end (sample) mode using default settings [2] with subsequent variant and consensus calling using VarScan 2.3.6 [3]. The subsequent genome was based on Prokka 1.8 [4] with subsequent manual curation for the strains JP48^{T} and JP55.

Supplementary Table 1. Substrate concentrations used for the determination of the substrate range of strains JP48^{T} and JP55 in liquid culture and on solid medium agar plates*.

Substrate	Concentration [mM]
Arabinose	5
Cellobiose	5
Erythrose	5
Erythrulose	5
Fructose	5
Fucose	5
Galactose	5
Glucose	5
Lactose	5
Lyxose	5
Maltose	5
Mannose	5
Melezitose	5
Raffinose	5
Rhamnose	5
Sorbose	5
Sucrose	5
Trehalose	5
Xylose	5
Glucosamine	5
N-acetylglucosamine	5
N-acetylgalactosamine	5
Acetoin	5
Adonitol	5
Arabitol	10
Dulcitol	5
Lyxitol	5
Mannitol	5
Myo-Inositol	5
Sorbitol	5
Xylitol	5
Alanine	5
Arginine	5
Asparagine	2
Aspartate	2
Cysteine	2
Glutamate	2
Glutamine	2
Glycine	5
Histidine	5
Hydroxy-Proline	5
Isoleucine	2
Leucine	5
Lysine	5
Methionine	5
Ornithine	2
Phenylalanine	5
Proline	2
Serine	2

Threonine	5
Tryptophan	1.25
Tyrosine	5
Valine	5
Adipate	5
Acetate	5
Ascorbate	5
Benzoate	5
Trimethoxybenzoate	5
Butyrate	2.5
α-Hydroxybutyrate	2.5
β-Hydroxybutyrate	2.5
γ-Hydroxybutyrate	2.5
Isobutyrate	2.5
Caproate	5
Caprylate	5
Citrate	2
Isocitrate	5
Crotonate	5
Formate	2.5
Fumarate	5
Gluconate	5
2-Oxogluconate	5
Glucuronate	5
2-Oxoglutarate	5
Glycolate	5
Glyoxylate	5
Heptanoic acid	5
Isovalerate	0.5
Laevulinate	5
Lactate	2
Malate	5
Maleic acid	5
Malonate	5
Nicotinic acid	2
Oxaloacetate	5
Propionate	5
Protocatechuate	5
Pyruvate	10
Shikimate	5
Succinate	10
Tartrate	2
2-Oxovalerate	5
Butanol	5
1,2-Butandiol	5
2,3-Butandiol	5
Ethanol	5
Ethylene glycol	5
Glycerol	5
Methanol	2
Propanol	5
1,2-Propandiol	5
Fermented rumen extract	5

Substrate	Concentration [% w/v]
Laminarin	0.05
Tween 80	0.001
Casamino acids	0.05
Casein hydrolysate	0.05
Peptone	0.05
**Substrate***	**Concentration [mg l^{-1}]**
Starch	500
Cellulose	500
Xylan	500
Polygalacturonic acid	500
Chitin	500
Pectin	500

Supplementary Table 2: Substrate degradation spectrum of JP48^T and JP55 in comparison to their phylogenetically related *Edaphobacter* species type strains.
Strains: 1, JP48^T; 2, JP55; 3, *Edaphobacter dinghuensis* DHF9^T [5]; 4, *Edaphobacter lichenicola* SBC68^T [6]; 5, *Edaphobacter modestus* Jbg-1^T [7]; 6, *Edaphobacter aggregans* Wbg-1^T [7]; 7, *Edaphobacter acidisoli* 4G-K17^T [8]; 8, *Edaphobacter bradus* 4MSH08^T [9]; 9*, Edaphobacter flagellatus* HZ411^T [9].
+, positive; -, negative; (+), weak growth detected; ND, no data available.

Carbon sources utilized	1	2	3	4	5	6	7	8	9
Arabinose	+	+	+	-	-	-	+	-	-
Cellobiose	+	+	+	+	-	-	+	-	+
Erythrose	(+)	(+)	ND	ND	-	-	ND	ND	ND
Erythrulose	(+)	(+)	ND	ND	-	-	ND	ND	ND
Fructose	+	+	+	+	+	-	+	+	+
Fucose	-	-	+	(+)	-	-	-	-	+
Galactose	+	+	+	+	-	-	+	-	+
Glucose	+	+	+	+	+	+	+	-	+
Lactose	+	+	+	+	+	+	+	+	+
Lyxose	-	-	-	ND	+	-	-	-	-
Maltose	+	+	+	+	-	-	+	+	+
Mannose	+	+	+	+	-	-	-	-	+
Melezitose	+	+	-	(+)	-	-	+	+	+
Raffinose	+	+	+	+	-	-	-	+	+
Rhamnose	+	+	+	+	+	-	+	-	+
Sorbose	-	-	-	-	-	-	+	-	+
Sucrose	+	+	+	+	ND	ND	ND	-	+
Trehalose	+	+	+	+	+	-	-	-	+
Xylose	+	+	+	+	+	-	-/+	-	-
Glucosamine	(+)	(+)	ND	ND	+	-	ND	ND	ND
N-acetyl-glucosamine	+	+	ND	(+)	-	-/+	+	+	+
N-acetyl-galactosamine	+	+	ND	ND	ND	ND	ND	ND	ND
Acetoin	(+)/+	(+)/+	ND	ND	-	-	ND	ND	ND
Adonitol	(+)	(+)	-	ND	ND	ND	-	-	-
Arabitol	-	-	-	ND	ND	ND	-	-	-
Dulcitol	(+)	(+)	-	-	-	-	-	-	-
Lyxitol	(+)	(+)	ND	ND	+	-	ND	ND	ND
Mannitol	-	-	-	(+)	+	-	-	+	-
Myo-Inositol	+	+	+	ND	+	-	-	+	+
Sorbitol	-	-	-	-	+	-	-	-	-
Xylitol	-	-	-	ND	+	-	-	-	-
Alanine	+	+	-	ND	-	-	ND	ND	ND
Arginine	+	+	ND	ND	-	-	ND	ND	ND
Asparagine	+	+	ND	ND	-	-	ND	ND	ND
Aspartate	+	+	ND	ND	-	+	-	ND	ND
Cysteine	(+)	(+)	ND	ND	-	-	ND	ND	ND
Glutamate	+	+	-	ND	+	+	ND	ND	ND
Glutamine	+	+	ND	ND	+	+	ND	ND	ND
Glycine	+	+	ND	ND	-	-	ND	ND	ND
Histidine	+	+	-	ND	-	-	ND	ND	ND
Hydroxy-Proline	-	-	ND	ND	ND	ND	ND	ND	ND
Isoleucine	+	+	ND	ND	-	-	ND	ND	ND
Leucine	+	+	ND	ND	-	-	ND	ND	ND
Lysine	-	-	ND	ND	-	-	ND	ND	ND
Methionine	+	+	ND	ND	-	-	ND	ND	ND
Ornithine	+	+	+	ND	-	+	ND	ND	ND
Phenylalanine	+	+	ND	ND	-	-	ND	ND	ND
Proline	+	+	ND	ND	-	-	ND	ND	ND
Serine	+	+	-	ND	-	-	ND	ND	ND

Threonine	+	+	ND	ND	-	-	ND	ND	ND
Tryptophan	+	+	ND	ND	-	-	ND	ND	ND
Tyrosine	+	+	ND	ND	-	-	ND	ND	ND
Valine	+	+	ND	ND	-	-	ND	ND	ND
Adipate	+	+	ND	ND	-	-	ND	ND	ND
Acetate	+	+	-	-	-	-	ND	ND	ND
Ascorbate	-/+	-/+	ND	ND	-	-	ND	ND	ND
Benzoate	(+)	(+)	-	ND	-	-	ND	ND	ND
Trimethoxybenzoate	+	+	ND	ND	-	-	ND	ND	ND
Butyrate	+	+	ND	-	-	-	ND	ND	ND
α-Hydroxy-butyrate	(+)	(+)	ND	ND	-	-	ND	ND	ND
β-Hydroxy-butyrate	+	+	ND	ND	-	-	ND	ND	ND
γ-Hydroxy-butyrate	+	+	ND	ND	-	-	ND	ND	ND
Isobutyrate	+	+	ND	ND	-	-	ND	ND	ND
Caproate	(+)	(+)	ND	ND	-	-	ND	ND	ND
Caprylate	(+)	(+)	ND	ND	-	-	ND	ND	ND
Citrate	+	+	-	-	-	-	-	ND	ND
Isocitrate	(+)	(+)	ND	ND	-	-	ND	ND	ND
Crotonate	(+)/+	(+)/+	ND	ND	-	-	ND	ND	ND
Formate	+	+	ND	-	-	-	ND	ND	ND
Fumarate	+	+	ND	(+)	-	-	ND	ND	ND
Gluconate	(+)	(+)	+	(+)	-	ND	-	-	-
2-Oxogluconate	+	+	ND	ND	-	-	ND	ND	ND
Glucuronate	-	-	ND	+	-	+	ND	ND	ND
2-Oxoglutarate	(+)/+	(+)/+	ND	ND	-	ND	ND	ND	ND
Glycolate	-	-	ND	ND	-	-	ND	ND	ND
Glyoxylate	-/+	-/+	ND	ND	-	-	ND	ND	ND
Heptanoic acid	+	+	ND	ND	-	-	ND	ND	ND
Isovaleric acid	+	+	ND	-	-	-	ND	ND	ND
Levulinate	-	-	ND	ND	-	-	ND	ND	ND
Lactate	-	-	-	(+)	-	-	ND	ND	ND
Malate	+	+	-/+	(+)	-	-	ND	ND	ND
Maleic acid	(+)	(+)	ND	ND	-	-	ND	ND	ND
Malonate	-	-	ND	ND	-	-	ND	ND	ND
Nicotinic acid	+	+	ND	ND	ND	ND	ND	ND	ND
Oxaloacetate	-	-	ND	ND	-	-	ND	ND	ND
Propionate	-	-	ND	-	-	-	ND	ND	ND
Protocatechuate	+	+	ND	ND	-	-	ND	ND	ND
Pyruvate	+/(+)	+/(+)	+	(+)	-	-	ND	ND	ND
Shikimate	+	+	ND	ND	-	-	ND	ND	ND
Succinate	+	+	+	+	-	-	-	ND	ND
Tartrate	+	+	(+)	ND	-	-	ND	ND	ND
2-Oxovalerate	(+)	(+)	ND	ND	-	-	ND	ND	ND
Butanol	-	-	ND	ND	-	-	ND	ND	ND
1,2-Butandiol	+	+	ND	ND	-	-	ND	ND	ND
2,3-Butandiol	+	+	ND	ND	-	-	ND	ND	ND
Ethanol	-	-	-	+	-	-	ND	ND	ND
Ethylene glycol	(+)	(+)	ND	ND	-	-	ND	ND	ND
Glycerol	+	+	-/+	ND	-	-	-	-	+
Methanol	-	-	-	-	-	-	ND	ND	ND
Propanol	-	-	ND	ND	-	-	ND	ND	ND
1,2-Propandiol	+	+	ND	ND	-	-	ND	ND	ND
Fermented rumen extract	+	+	ND	ND	-	-	ND	ND	ND
Tween 80	+	+	ND	ND	ND	ND	ND	ND	ND
Casamino acids	+	+	+	ND	+	+	ND	ND	ND
Casein hydrolysate	+	+	ND	ND	ND	ND	ND	ND	ND
Peptone	+	+	ND	ND	+	+	ND	ND	ND
Yeast extract	+	+	+	ND	+	+	ND	ND	ND
Laminarin	+	+	ND	+	ND	ND	ND	ND	ND
Chitin	-	-	ND	-	ND	ND	ND	ND	ND

Cellulose	-	-	ND	-	ND	ND	ND	ND	ND
Pectin	-	-	ND	-	ND	ND	ND	ND	ND
Starch	-	(+)	+	-	ND	ND	+	-	+
Xylan	-	-	ND	+	ND	ND	ND	ND	ND

Supplementary Table 3: Api®ZYM and API®20NE test spectrum of JP48^{T} and JP55 in comparison to their phylogenetically related *Edaphobacter* species type strains.
Strains: 1, JP48^{T}; 2, JP55; 3, *Edaphobacter dinghuensis* DSM 29920^{T}; 4, *Edaphobacter lichenicola* DSM 104462^{T}, 5, *Edaphobacter modestus* Jbg-1^{T} [7]; 6, *Edaphobacter aggregans* Wbg-1^{T} [7]; 7, *Edaphobacter acidisoli* 4G-K17^{T} [8]; 8, *Edaphobacter bradus* 4MSH08^{T} [9]; 9, *Edaphobacter flagellatus* HZ411^{T} [9].
+, positive; -, negative; (+), weak growth detected; ND, no data available.

**Characteristics**	**1**	**2**	**3[a]**	**4[a]**	**5**	**6**	**7**	**8**	**9**
Alkaline phosphatase	-	-	-	+	+	+	+	+	+
Esterase C4	-	-	(+)	(+)	+	+	-	-	+
Esterase lipase C8	-	-	-	(+)	+	+	+	-	+
Lipase C14	-	-	-	-	-	-	-	-	-
Leucine arylamidase	-	-	-	+	+	+	+	+	+
Valine arylamidase	-	-	-	+	+	+	+	+	+
Cysteine arylamidase	-	-	-	(+)	-	-	+	+	+
Trypsin	-	-	-	-	-	-	+	-	-
α-Chymotrypsin	-	-	-	(+)	+	+	+	-	+
Acid phosphatase	+	+	+	+	+	+	+	-	+
Naphtol-AS-BI-phosphohydrolase	+	(+)	+	+	+	+	+	+	+
α-Galactosidase	+	+	-	-	+	+	+	+	+
β-Galactosidase	+	+	+	+	+	+	+	+	+
β-Glucuronidase	+	(+)	(+)	+	-	-	+	+	-
α-Glucosidase	+	+	-	-	+	+	+	+	+
β-Glucosidase	+	+	-	+	+	+	+	+	+
N-Acetylglucosaminidase	+	+	(+)	+	-	-	+	+	+
α-Mannosidase	-	-	-	-	-	-	+	+	+
α-Fucosidase	+	+	-	-	-	-	+	+	+
Nitrate reduction	+	(+)	+	-	-[a]	-[a]	-	-	-
β-Glucosidase	+	+	+	+	+[a]	+[a]	+	+	+
β-Galactosidase	+	+	+	+	+[a]	+[a]	+	+	+
Urease	-	-	-	-	-	-	+	+	+

[a] Data obtained in the present study.

Supplementary Table 4. Summary of genomic features of the strains JP48^T, JP55.

	**JP48^T**	**JP55**
**Genome length [bp]**	4,038,441	4,316,569
**G + C content [mol%]**	57.4	57.2
**sequencing coverage values**	1764	1966
**CDS regions**	3412	3696
**rRNA**	3	3
**tRNA**	48	48

Supplementary Table 5. Overview of the 16S rRNA gene sequence and whole-genome-similarity indices of the strains JP48^T and JP55 compared with their phylogenetically most closely related type strains *Edaphobacter dinghuensis* CGMCC 1.12997^T [5] and *Edaphobacter lichenicola* DSM 104462^T [6].

	***Edaphobacter dinghuensis* CGMCC 1.12997^T**	***Edaphobacter lichenicola* DSM 104462^T**
	**16S rRNA**	
**JP48^T**	98.3	96.9
**JP55**	98.3	96.9
	**ANI**	
**JP48^T**	80.7	74.3
**JP55**	80.8	74.3
	**dDDH**	
**JP48^T**	24.2	20.3
**JP55**	24.2	20.3
	**AAI**	
**JP48^T**	83.9	71.1
**JP55**	84.2	71.4

Supplementary Table 6. Overview of antiSMASH analysis results of the strains JP48^T, JP55 and their phylogenetically most closely related type strains *Edaphobacter dinghuensis* CGMCC 1.12997^T [5] and *Edaphobacter lichenicola* DSM 104462^T.

**Strain**		**JP48^T and JP55**	
**Genome sequence**		**CP121194 and CP121195**	
**Region**	**Type**	**Most similar known cluster**	**Similarity (%)**
1	T3PKS	ambruticin - Polyketide	5
2	NRPS-like, T1PKS, NRPS	jamaicamide A/jamaicamide B/jamaicamide C - NRP+Polyketide	7
3	NRPS-like	-	-
4	terpene	-	-
5	NRPS-like	-	-
6	lanthipeptide-class-iv	-	-
7	terpene	-	-
8	T3PKS	nosiheptide - RiPP:Thiopeptide	11
9	T3PKS	-	-
**Strain**		***Edaphobacter dinghuensis* CGMCC 1.12997^T**	
**Genome sequence**		**BMGT01000001.1**	
1.1	T3PKS	-	-
**Genome sequence**		**BMGT01000002.1**	
2.1	NRPS-like	-	-
2.2	terpene	-	-
2.3	T3PKS	-	-
**Genome sequence**		**BMGT01000003.1**	
3.1	RiPP-like	-	-
3.2	terpene	-	-
		**BMGT01000004.1**	
4.1	NRPS, RRE-containing, lassopeptide	endopyrrole B/endopyrrole A - NRP:Cyclic depsipeptide	50
**Genome sequence**		**BMGT01000005.1**	
5.1	NRPS	xenoamicin A/xenoamicin B - NRP:Cyclic depsipeptide	20
**Strain**		***Edaphobacter lichenicola* DSM 104462^T**	
**Genome sequence**		**GCA_025264645.1**	
**1**	terpene, NRPS-like		
**2**	RiPP-like		
**3**	terpene		
**4**	redox-cofactor		
**5**	NRPS, T1PKS, RiPP-like		
**6**	terpene		
**7**	NRPS-like		
**8**	T3PKS		

Supplementary Table 7. Intact polar lipids (IPL) of JP48^T and JP55 compared to their closest relatives.
Strains: 1, JP48^T; 2, JP55; 3, *Edaphobacter dinghuensis* DSM 29920^T; 4, *E. lichenicola* DSM 104462^T; 5, *E. aggregans* DSM 19364^T; 6, *E. modestus* DSM 18101^T.

The strains were grown according their optimum growth conditions. In detail: strains 1, 2 and 3 on DSMZ medium 1426 at 28°C, strain 4 on DSMZ medium 1284 at 25°C, strain 5 on DSMZ medium 1135 at 20°C, and strain 6 on DSMZ medium 1124 at 25°C.

Intact Polar Lipids	1	2	3	4	5	6
Phosphatidylethanolamine	+	+	+	+	+	+
Diphosphatidylglycerol	+	+	+	+	+	+
Phosphatidylglycerol	+	+	+	+	-	-
Lysophosphatidylethanolamine	+	+	-	+	+	-
Phosphatidylcholine	+	+	+	+	+	+
Ornithine lipid	-	-	-	-	+	-
Glycophospholipid	+	+	+	+	+	+
Glycolipid	+	+	-	+	+	+
Unidentified high masses	+	+	+	+	+	+

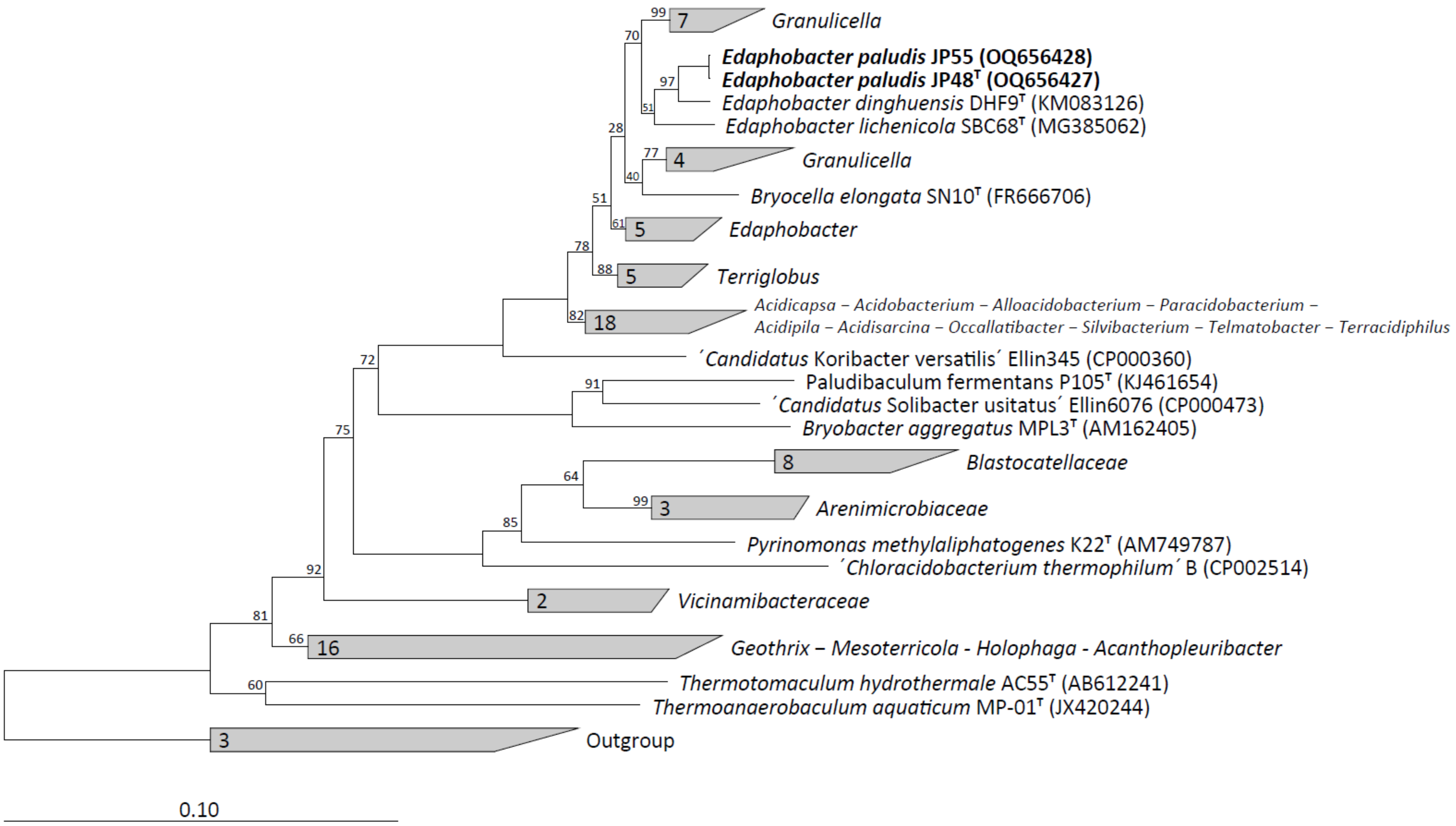

Supplementary Figure 1.

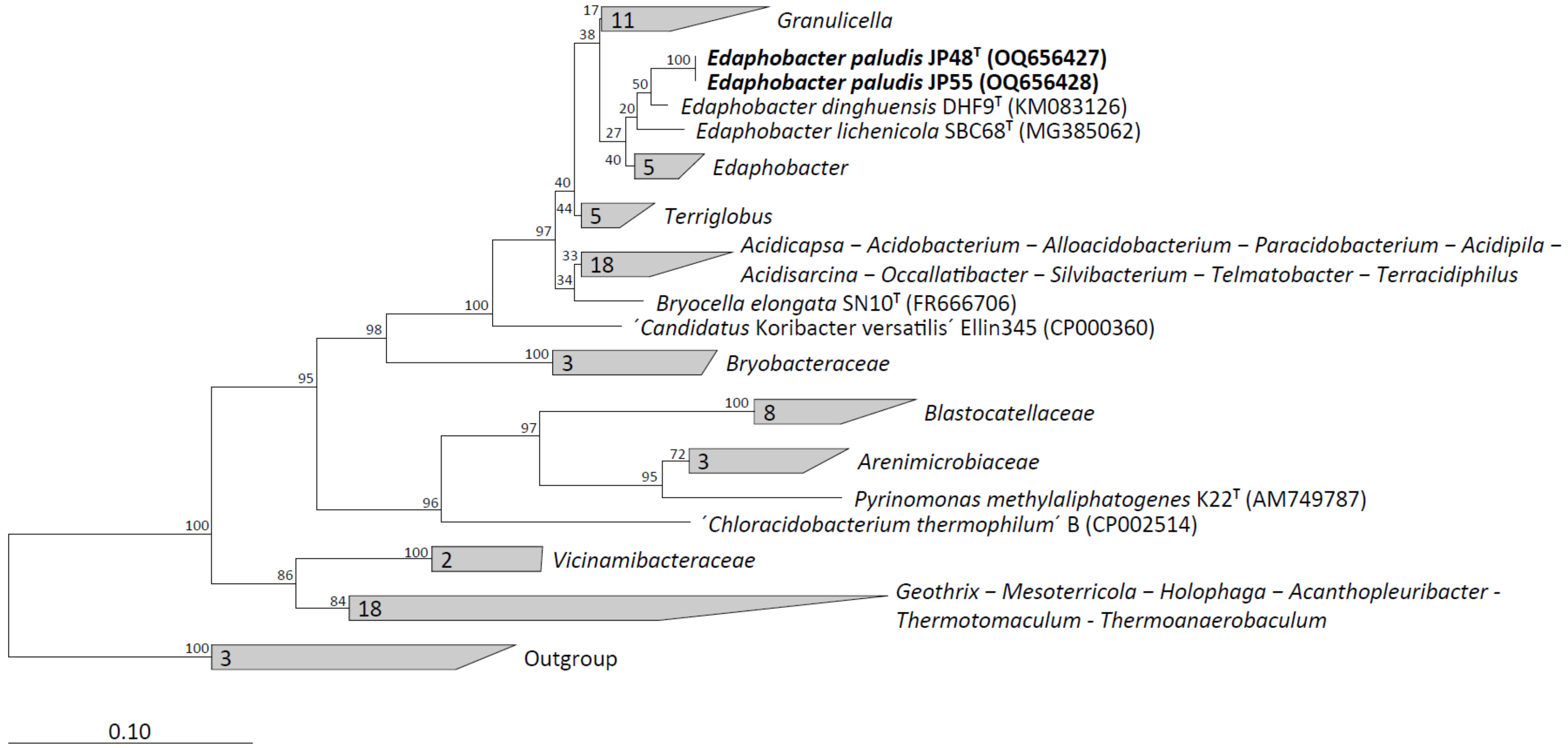

Supplementary Figure 2.

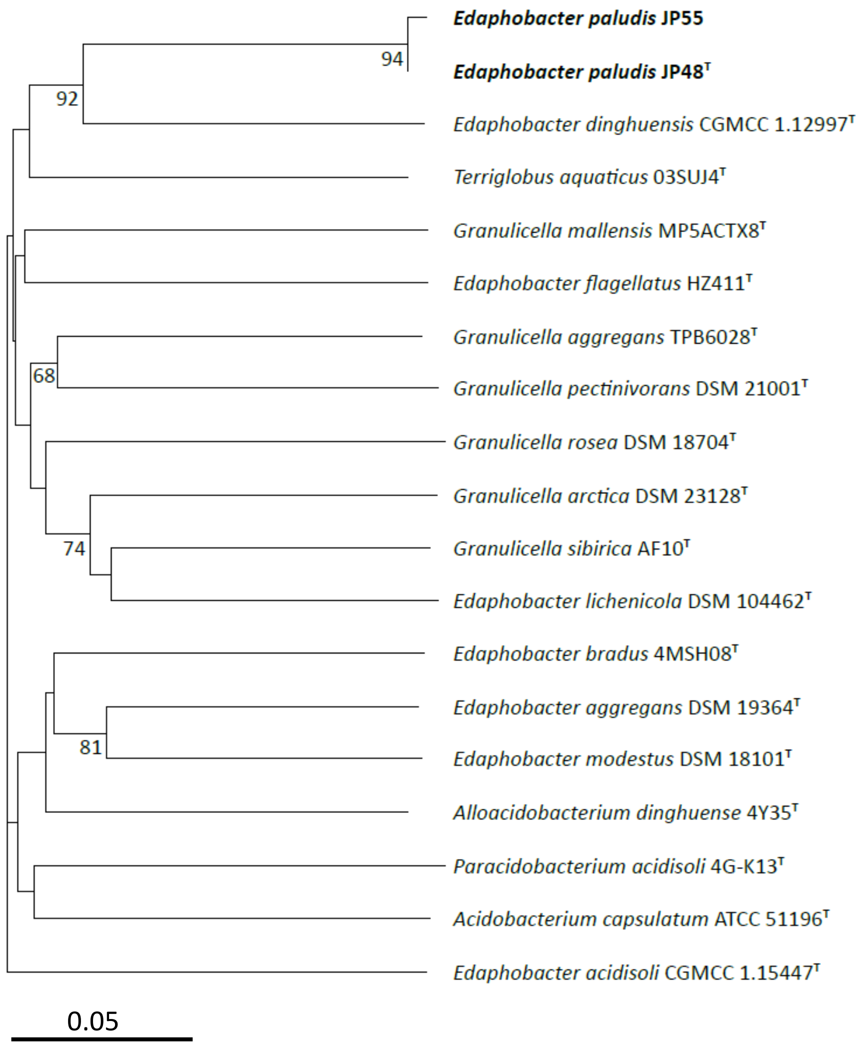

Supplementary Figure 3.

FIGURE LEGENDS

Supplementary Figure 1. Rooted neighbor-joining phylogenetic tree (Felsenstein correction) based on almost full-length 16S rRNA gene sequences showing the relationship of the strains JP48^T, JP55 and related type strains. Bootstrap values are expressed as percentages of 1000 replicates and are indicated at the respective branching points. The following sequences were used as outgroup: *Novipirellula rosea* LHWP3^T (JF748734), *Blastopirellula marina* DSM 3645^T (X62912) and *Pirellula staleyi* DSM 6068^T (CP001848). Bar indicates 10% nucleotide divergence.

Supplementary Figure 2. Maximum-Parsimony phylogenetic tree based on almost full-length 16S rRNA gene sequences showing the relationship of the strains JP48^T, JP55 and related type strains. Bootstrap values are expressed as a percentages of 1000 replicates and are indicated at the respective branching points. The following sequences were used as outgroup: *Novipirellula rosea* LHWP3^T (JF748734), *Blastopirellula marina* DSM 3645^T (X62912) and *Pirellula staleyi* DSM 6068^T (CP001848). Bar indicates 10% nucleotide divergence.

Supplementary Figure 3. Whole-genome-based GBDP distances tree for the strains JP48T and JP55. The tree was inferred with FastME 2.1.6.1 [10] from GBDP distances calculated from genome sequences. The branch lengths are scaled in terms of GBDP distance formula d5. The numbers above branches are GBDP pseudo-bootstrap support values > 60 % from 100 replications, with an average branch support of 47.0 %. The tree was rooted at the midpoint [11].

REFERENCES

1. **Baym M, Kryazhimskiy S, Lieberman TD, Chung H, Desai MM, Kishony R**. Inexpensive multiplexed library preparation for megabase-sized genomes. *PLoS One* 2015;10:e0128036.
2. **Li H, Durbin R.** Fast and accurate short read alignment with Burrows-Wheeler transform. *Bioinformatics* 2009;25:1754-60.
3. **Koboldt DC, Zhang Q, Larson DE, Shen D, McLellan MD *et al.*** VarScan 2: somatic mutation and copy number alteration discovery in cancer by exome sequencing. *Genome Res* 2012;22:568-76.
4. **Seemann T.** Prokka: rapid prokaryotic genome annotation. *Bioinformatics* 2014;30:2068-9.
5. **Wang J, Chen MH, Lv YY, Jiang YW, Qiu LH.** *Edaphobacter dinghuensis* sp. nov., an acidobacterium isolated from lower subtropical forest soil. *Int J Syst Evol Microbiol* 2016;66:276-282.
6. **Belova SE, Suzina NE, Rijpstra WIC, Sinninghe Damste JS, Dedysh SN.** *Edaphobacter lichenicola* sp. nov., a member of the family *Acidobacteriaceae* from lichen-dominated forested tundra. *Int J Syst Evol Microbiol* 2018;68:1265-1270.
7. **Koch IH, Gich F, Dunfield PF, Overmann J**. *Edaphobacter modestus* gen. nov., sp. nov., and *Edaphobacter aggregans* sp. nov., acidobacteria isolated from alpine and forest soils. *Int J Syst Evol Microbiol* 2008;58:1114-1122.
8. **Xia F, Cai YM, Chen DX, Qiu LH.** *Edaphobacter acidisoli* sp. nov., an acidobacterium isolated from forest soil. *Int J Syst Evol Microbiol* 2017;67:4260-4265.
9. **Xia F, Ou-Yang TN, Gao ZH, Qiu LH.** *Edaphobacter flagellatus* sp. nov. and *Edaphobacter bradus* sp. nov., two acidobacteria isolated from forest soil. *Int J Syst Evol Microbiol* 2018;68:2530-2537.
10. **Lefort V, Desper R, Gascuel O**. FastME 2.0: A comprehensive, accurate, and fast distance-based phylogeny inference program. Mol Biol Evol. 2015;32: 2798–2800.
11. **Farris JS**. Estimating phylogenetic trees from distance matrices. *Am Nat* 1972;106: 645–667.
